# Selective Targeting of IL-15Rα Is Sufficient to Reduce Inflammation

**DOI:** 10.3389/fimmu.2022.886213

**Published:** 2022-05-03

**Authors:** Dihia Meghnem, Mike Maillasson, Isabelle Barbieux, Sébastien Morisseau, Dalloba Keita, Yannick Jacques, Agnès Quéméner, Erwan Mortier

**Affiliations:** ^1^ Nantes University, CNRS, Inserm, CRCI2NA, Nantes, France; ^2^ LabEX IGO, Immuno-Onco-Greffe, Nantes, France; ^3^ Nantes University, Centre Hospitalo-Universitaire (CHU) Nantes, Inserm, CNRS, SFR Bonamy, UMS BioCore, IMPACT Platform, Nantes, France; ^4^ Centre Hospitalo-Universitaire (CHU), Nantes Hospital, Nantes, France

**Keywords:** interleukin, receptor, IL-2, inhibition, homeostasis

## Abstract

Cytokines are crucial molecules for maintaining the proper functioning of the immune system. Nevertheless, a dysregulation of cytokine expression could be involved in the pathogenesis of autoimmune diseases. Interleukin (IL)-15 is a key factor for natural killer cells (NK) and CD8 T cells homeostasis, necessary to fight cancer and infections but could also be considered as a pro-inflammatory cytokine involved in autoimmune inflammatory disease, including rheumatoid arthritis, psoriasis, along with tumor necrosis factor alpha (TNF-α), IL-6, and IL-1β. The molecular mechanisms by which IL-15 exerts its inflammatory function in these diseases are still unclear. In this study, we generated an IL-15-derived molecule called NANTIL-15 (New ANTagonist of IL-15), designed to selectively inhibit the action of IL-15 through the high-affinity trimeric IL-15Rα/IL-2Rβ/γc receptor while leaving IL-15 signaling through the dimeric IL-2Rβ/γc receptor unaffected. Administrating of NANTIL-15 in healthy mice did not affect the IL-15-dependent cell populations such as NK and CD8 T cells. In contrast, we found that NANTIL-15 efficiently reduced signs of inflammation in a collagen-induced arthritis model. These observations demonstrate that the inflammatory properties of IL-15 are linked to its action through the trimeric IL-15Rα/IL-2Rβ/γc receptor, highlighting the interest of selectively targeting this receptor.

## Introduction

Cytokines are key mediators involved in the regulation of the normal immune response. Dysregulation of cytokine expression plays a complex role in the pathogenesis of autoimmune diseases. Interleukin (IL)-15, a cytokine discovered in 1994, belongs to the cytokine family sharing the common γ (γc) receptor chain (CD132), including IL-2, IL-4, IL-7, IL-9, IL-15, and IL-21 (1). Among them, IL-2 and IL-15 share IL-2Rβ (CD122) and γc receptor chains that form a common heterodimeric IL-2Rβ/γc receptor. Thus, IL-2 and IL-15 are redundant cytokines with common functions *in vitro*, such as T- and NK-cell proliferation (2, 3). However, *in vivo*, IL-2- and IL-15-deficient mice show relatively distinct phenotypes. Indeed, IL-2-deficient mice exhibit spontaneous T-cell accumulation, indicating that IL-2 *in vivo* restrains T-cell activation (4, 5), whereas IL-15-deficient mice lack NK cells, CD8 memory T cells, NK-T cells, and subset of IELs, indicating that IL-15 is essential for the development of these cells (6). The specificity of action of IL-2 and IL-15 is conferred by their alpha receptor chains, IL-2Rα (CD25) and IL-15Rα (CD215) (7, 8), respectively, forming a high-affinity heterotrimeric receptor with IL-2Rβ and γc chains, making cells more sensitive to low concentrations of cytokines compared to the dimeric receptor. Signaling of IL-2 or IL-15 by the dimeric IL-2Rβ/γc and trimeric IL-2Rα/or IL-15Rα/IL-2Rβ/γc receptor only differs in the kinetics of activation (9), recruiting relatively similar downstream signaling pathways (10). Interestingly, the phenotype of mice deficient for IL-2Rα and IL-15Rα resembles that of mice deficient for the corresponding cytokine (11–13), revealing that the actions of these cytokines are closely related to the expression of their private alpha receptor chain. IL-2Rα is constitutively expressed by regulatory T cells and activated T cells, whereas IL-15Rα can be more broadly expressed independently of IL-2Rβ and γc chains. IL-2Rα binds IL-2 with a low affinity (Kd = 10 nM), whereas IL-15Rα binds to IL-15 with high affinity (Kd = 50 pM), retaining IL-15 on the cell surface (10). Interestingly, IL-15 and IL-15Rα can pre-associated within the producing cells before emerging to the cell surface (14). At the cell surface, IL-15Rα is able to present IL-15 in *trans* to dimeric IL-2Rβ/γc receptors on nearby effector NK and T cells by the formation of an immunological synapse (15). This immunological synapse is thought to limit exposure to circulating IL-15, in which uncontrolled expression would undeniably lead to the induction of autoimmunity. IL-15 expression is highly regulated, and circulating free IL-15 is difficult to detect in fluids and cell culture supernatants (14) but rather exists in a heterodimeric form associated with soluble IL-15Rα (16). Nevertheless, dysregulation of IL-15 expression with concomitant elevated levels of IL-15 has been reported in several autoimmune diseases (17, 18), including rheumatoid arthritis (19), psoriasis (20), lupus (21), sarcoidosis (22, 23), type 1 diabetes (24), celiac disease (25), and inflammatory bowel diseases (26). For these reasons, IL-15 has been considered as a key cytokine sitting at the apex of a pyramid of the pro-inflammatory cytokines (27).

Owing to its clear involvement in these pathologies, a series of IL-15-directed antagonistic approaches have been developed to limit aberrant immune stimulation and decrease the risk of autoimmunity related to uncontrolled IL-15 exposure. The use of soluble forms of IL-15Rα (28), IL-15 mutants (29), monoclonal antibodies directed against IL-15 or IL-15 receptor (20, 30, 31), peptides (32, 33), and small chemical compounds (34–36) have been shown to be relatively effective in inhibiting IL-15 action *in vitro*. In addition, some of them showed their efficacy to improve the clinical signs of inflammation in animal models (37). Based on the observations of Pettit et al., the molecule CRB-15, bearing Q101D and Q108D mutations located in the region of IL-15 binding to γc receptor chain, was designed to selectively and competitively block IL-15-triggered cell proliferation by impairing the recruitment of the γc chain receptor (29, 38). Administration of CRB-15, or a soluble form of IL-15Rα chain, profoundly suppressed the development of collagen-induced arthritis (39, 40). Moreover, CRB-15 treatment also improved the survival of DSS-challenged mice in a chronic colitis model, and soluble IL-15Rα also prevented psoriasis (41) and allergic inflammation (42). In this context, Amgen has developed an anti-IL-15 antibody (AMG-714 or HuMax-IL15) targeting IL-15 in the region involved in γc chain binding. AMG-714 first showed efficacy in a xenograft model of human psoriasis and rheumatoid arthritis (19) and coeliac disease (43). Another approach has been recently developed by designing antagonistic peptides, called BNZ, targeting the γc receptor chain, acting as multicytokine inhibitors (32, 44). A first-in-human study showed that BNZ-1 (IL-2/9/15 inhibitor) could be safely administrated with robust and reversible immunomodulation (45). A phase I/II clinical trial involving BNZ-1-targeted T-cell malignancies CTCL and LGLL has been completed (NCT03239392), and the efficacy of BNZ-1 and BNZ-2 (IL-15/21) is currently being evaluated in alopecia areata and refractory coeliac disease, respectively (46).

**Figure 1:**
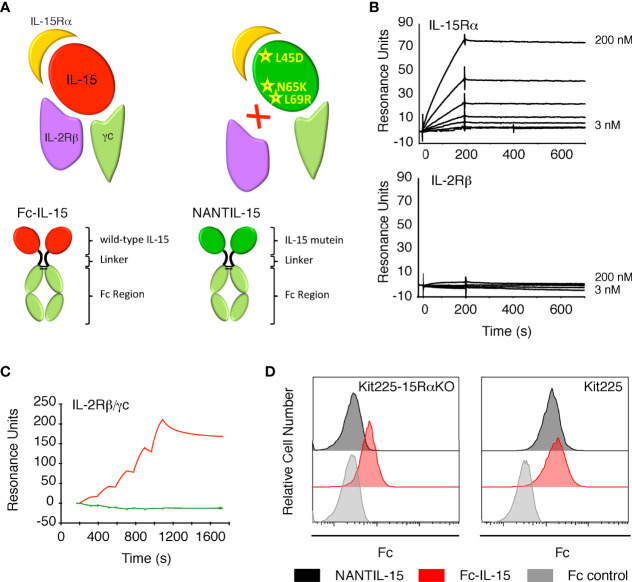
Binding of NANTIL-15 on IL-15 receptors. **(A)** Top panels: diagram showing wild-type IL-15 (red) or IL-15 mutein (green) bound to its three receptor chains IL-15Rα, IL-2Rβ, and γc. Bottom panels show the Fc-IL-15 and NANTIL-15 molecules. **(B)** SPR sensorgrams of the binding of increasing concentrations of NANTIL-15 to immobilized IL-15Rα (upper panel) or IL-2Rβ (lower panel) chains. **(C)** SPR sensorgram of the binding of increasing concentrations of NANTIL-15 (green) or Fc-IL-15 (red) to immobilized Fc-IL-2Rβ/γc proteins. **(D)** Binding of NANTIL-15 on Kit225 (expressing IL-15Rα, IL-2Rβ, and γc) or Kit225-15RαKO (expressing IL-2Rβ and γc) cells, revealed by flow cytometry. All data are representative of at least three separate experiments.

Although these strategies are effective in blocking the action of IL-15, the molecular mechanism by which IL-15 drives inflammation remains unclear. In the present study, we hypothesized that retaining the binding to IL-15Rα while not recruiting IL-2Rβ would block IL-15’s signaling through the trimeric IL-15Rα/IL-2Rβ/γc receptor without affecting the one through the dimeric IL-2Rβ/γc receptor. We previously identified an interface of IL-15 facing IL-2Rβ as being crucial for the binding of IL-15 to IL-2Rβ (47, 48). This interface includes the residues N65 and L69, and based on these previous observations, we generated an IL-15 mutein in which these residues were exchanged by K and R residues, respectively, to fully abrogate the recruitment of IL-2Rβ. The mutation L45D was also added to increase IL-15’s production in our expression system. In addition, an Fc portion has been grafted to the IL-15 triple mutant to enhance its inhibitory properties of IL-15’s action *in vivo* by increasing the half-life of the molecule. This molecule was called NANTIL-15 (New ANTagonist of IL-15). We also generated a similar fusion molecule with wild-type IL-15, namely, Fc-IL-15, which we used as control (**Figure 1A**). In this study, we characterized the inhibitory properties of NANTIL-15 *in vitro*. Then, we tested the impact of NANTIL-15 administration in mice on IL-15-dependent cells, such as NK and CD8 T cells. Finally, the efficacy of NANTIL-15 in reducing inflammation was evaluated in a collagen-induced arthritis model.

## Materials and Methods

### Cytokines and Antibodies

Antibodies for flow cytometry were purchased from BD Biosciences or eBioscience. The CF1 antibody directed against a non-neutralizing epitope of IL-2Rβ was obtained from Beckman Coulter (Villepinte, France) and the blocking A41 anti-IL-2Rβ antibody, produced in house, has been previously characterized (49, 50). The NANTIL-15 molecules correspond to human IL-15 bearing the triple mutation L45D-N65K-L69R and linked to either a human Fc (IgG1) for *in vitro* experiments or a murine Fc (IgG2a) for *in vivo* experiments.

Recombinant 6xHis tagged human IL-15, IL-15Rα-Linker-IL-15 (RLI), IL-15_D8S_, NANTIL-15, and Fc-IL-15 were produced by Evitria (Schlieren, Switzerland) and purified by HIS TRAP Excel column using an AKTA start system (Cytiva, Versailles, France). Fc-IL-15Rα and recombinant human IL-2Rβ were obtained from R&D Systems (Lille, France) Fc-IL-2Rβ/γc was kindly provided by Cytune Pharma (Nantes, France) and was produced using a Knobs-In-Holes strategy from CHO supernatant and purified on protein A, Nuvia HR-S, and gel filtration by Evitria (Switzerland). Recombinant human IL-2 was purchased from Chiron (Emeryville, CA, USA).

### Cell Culture

HEK-293 cell line was cultured in Dulbecco’s modified Eagle’s medium (DMEM) medium (Thermo Fisher, Illkirch, France) containing 10% of heat-inactivated fetal calf serum (FCS) (Gibco Illkirch, France), 2 mM glutamine, and 1 mg/ml glucose. Stably transfected HEK-293 cells expressing membrane-bound IL-15Rα (IL-15Rα-HEK-293 cells) (51) were cultured in the same medium supplemented with 50 ng/ml puromycin. Kit225 and Kit225-15RαKO (48) cells were cultured as described previously (51). The CTLL-2 mouse cytotoxic T-lymphocyte cell line was cultured in Roswell Park Memorial Institute (RPMI)-1640 medium (Sigma-Aldrich, Saint-Quentin-Fallavier, France) containing 8% heat-inactivated FCS, 2 mM glutamine, 10 µM 2-mercaptoethanol, and 15 ng/ml human rIL-2. All cell lines were maintained at 37°C, in a humidified, 5% CO_2_ atmosphere.

### Mice

C57BL/6 and DBA/1 mice were purchased from the Janvier Labs (Le Genest St. Isle, France). Mice were generally used between the age of 6 and 12 weeks. The procedures were approved by the French Ethics Committee for Animal Experimentation and performed according to European Union guidelines.

### ELISA Assay

NANTIL-15 plasma levels were evaluated by ELISA using a human Fc-IL-15Rα (R&D Systems) as capture and an horseradish peroxidase (HRP) goat anti-mouse IgG (H+L) (INTERCHIM, Montluçon, France) as the revealing antibody. A standard curve was performed with NANTIL-15.

### SPR Assays

The biosensor used in this study was a Biacore T200 instrument (Cytiva). CM5 Research Grade Sensor Chips (carboxy-methyl-dextran derivatized with carboxyl group) and HBS-EP [0.01M HEPES, pH 7.4, 0.15M NaCl, 0.005% (v/v) surfactant P20, and 3 mM EDTA] running buffer were purchased from Cytiva. All surface plasmon resonance (SPR) experiments were performed at a flowrate of 40 µl/min at 25°C. Fc-IL-15Rα, recombinant human IL-2Rβ, or anti-IL-2Rβ CF1 antibody was coupled onto the CM5 surface following the standard amine coupling protocol (according to supplier’s procedures) to achieve a residual coupling response of 300, 250, and 8,000 RU, respectively.

*SPR Kinetic Analysis on Fc-IL-15Rα and Recombinant Human IL-2Rβ.* Recombinant NANTIL-15 protein was diluted in HBS-EP at concentrations ranging from 3.12 to 200 nM and injected over the Fc-IL-15Rα or the IL-2Rβ immobilized surface in a kinetic mode. Association and dissociation were monitored for 3 and 10 min, respectively. A 10 mM Glycin pH 1.5 solution was injected over the chips for 30 s for regeneration between each cycle. Rmax value (RU), k_on_ (M^−1^ s^−1^), k_off_ (s^−1^), and Kd (M) were calculated from kinetics sensorgrams using the Langmuir 1:1 model.

*SPR Kinetic Analysis on Recombinant Dimeric Fc-IL-2Rβ/γc Receptor.* A capture strategy was used for SPR analysis on Fc-IL-2Rβ/γ. Briefly, recombinant Fc-IL-2Rβ/γc diluted at 200 nM in HBS-EP was injected over the CF1 immobilized chip for 1 min. After a 1-min stabilizing period, NANTIL-15 or Fc-IL-15 diluted in HBS-EP at concentrations ranging from 31.5 to 500 nM were injected over the Fc-IL-2Rβ/γ captured surface in a Single Cycle Kinetics (SCK) mode. Association and dissociation were monitored for 2 and 10 min, respectively. A 10 mM Glycin pH 1.5 solution was injected over the chips for 30 s for regeneration between each capture cycle. Rmax value (RU), k_on_ (M^−1^ s^−1^), k_off_ (s^−1^), and Kd (M) were calculated from Single Cycle kinetics sensorgrams using the Langmuir 1:1 model.

### Proliferation Assays

The proliferation of Kit225, Kit225-15RαKO, and CTLL-2 cells in response to IL-2, IL-15, IL-15_D8S_, and RLI was assessed by Alamar blue reduction assay (AbDSerotec, Marnes-la-Coquette, France). Cells were starved in the culture medium without cytokine for 4 h in case of CTLL-2 cells to reduce background or overnight in case of Kit225 and Kit225-15RαKO cells. Viability of cells was controlled by trypan blue staining. Then, cells (1 × 10^4^) were preincubated for 30 min with increasing concentrations of NANTIL-15 and then cultured for 2.5 days in the medium supplemented with IL-2 (100 pM), IL-15 (10 and 100 pM), IL-15_D8S_ (10 and 100 pM), or RLI (100 pM). Cell proliferation was revealed by adding Alamar blue (10 µl) to each well and, after a 6-h incubation period at 37°C, by measuring the emitted fluorescence at 590 nm under excitation at 560 nm using an EnSpire Multimode Plate Reader (PerkinElmer, Courtaboeuf, France).

### pStat5 Assays by Flow Cytometry and AlphaScreen Technology

Detection of pStat5 was carried out using either flow cytometry or the AlphaLISA SureFire Ultra™ assay kit (PerkinElmer). For both assays, exponentially growing Kit225 or Kit225-15RαKO cells were washed and starved overnight in the culture medium without cytokines to reduce basal phosphorylation. Cells were preincubated for 30 min with a fixed or increasing concentrations of NANTIL-15 or A41 anti-IL-2Rβ antibody and then stimulated at 37°C for 1 h with fixed concentrations of IL-15, RLI, or IL-15 mutants. For IL-15 *trans*-presentation experiments, Kit225 cells labeled with BD Horizon Violet Proliferation dye 450 (VPD-450; BD Biosciences, Le Pont de Chaix, France) were cocultured for 1 h with IL-15Rα HEK-293 cells preloaded for 30 min with IL-15.

For flow cytometry pStat5 staining, 3 × 10^5^ stimulated cells were fixed for 15 min at 37°C using Fix Buffer I (BD Biosciences) and permeabilized in Perm Buffer III (BD Biosciences) for 20 min at 4°C. Then, cells were labeled with phycoerythrin (PE)-conjugated mouse anti-human Stat5 (Y694) or PE-conjugated control isotype (BD Biosciences) for 1 h at room temperature. Cells were analyzed using BD FACS Calibur, or BD FACS Canto 2 (BD Biosciences) and FlowJo software.

For AlphaLISA assay, 2 × 10^5^ stimulated cells were lysed, and Stat5 phosphorylation was measured according to the manufacturer’s instructions using pStat5 AlphaScreen Surefire kit. Then, the signal in the wells was detected using an EnSpire Multimode Plate Reader (PerkinElmer).

### Pharmacokinetic Studies

Bioavailability of NANTIL-15 was evaluated in male C57BL/6 mice after a single intravenous injection of 10 µg of NANTIL-15. At each time point following injection, blood was collected (three mice per point) and centrifugated, and the plasma was frozen at −20°C. NANTIL-15 plasma levels were measured by ELISA (see above). Pharmacokinetic parameters were calculated using a one-compartment model with GraphPad Prism software.

### *In Vivo* Evaluation of NANTIL-15 Administration on NK and CD8 T-Cell Homeostasis

The effect of repeated injections of NANTIL-15 was evaluated *in vivo* in male C57BL/6 mice. Mice received one daily i.p. injection of 1.5, 5, or 25 µg of NANTIL-15 or phosphate-buffered saline (PBS) over 4 or 14 days. The day following the last injection, mice were sacrificed, and spleens were collected for NK and CD8^+^ T cells by flow cytometric analysis.

### Murine Collagen-Induced Arthritis Model

Male DBA/1 mice 6–8 weeks of age (Janvier Labs, Le Genest St. Isle, France) were used in all experiments. Type II collagen from bovine tracheal cartilage (Sigma C1188) was dissolved in 0.1M acetic acid at 2 mg/ml at 4°C overnight. The collagen solution was then emulsified using a homogenizer with a small blade with an equal volume of Freund’s complete adjuvant (Difco 231131) as previously described (40). To induce CIA, 200 µg of collagen was injected intradermally at the base of the tail of DBA/1 mice. Twenty-one days after immunization, the animals were challenged with 200 µg of collagen i.p. in 0.05M acetic acid. Mice were monitored every day to observe the apparition of clinical signs of arthritis based on the following criteria: grade 0, normal joints and no swelling; grade 1, mild swelling and/or erythema; grade 2, pronounced edema or redness of the paw or several digits; and grade 3, severe swelling of entire paw and/or ankylosis. As each limb was individually graded, the maximal clinical score for each mouse was 12. The thickness of each paw was also measured using a caliper, and the mean paw thickness was calculated. Administration of NANTIL-15 or IgG2a (eBioscience 14-4724) treatment (daily i.p. injection at 1.5 or 5 µg/injection/day for 14 days, based on 39) was either started in a randomized manner after the establishment of the arthritis (i.e., clinical score at least equal to 1) or 1 day after the second immunization. Spleens were collected 1 h after the last injection of NANTIL-15 for flow cytometric analysis of NK and CD8 T cells. Paws were removed post-mortem, fixed in 1% paraformaldehyde for 3 days, and decalcified in 5% EDTA for 2 weeks before being embedded in paraffin for sectioning. Serial sagittal sections were made, mounted on glass slides, and stained with H&E and for CD8 marker. Tissue sections were analyzed by light microscopy.

### Flow Cytometric Analysis of *Ex Vivo* NK and CD8 T Cells

Single-cell suspensions from spleen cleared from RBCs were obtained and stained. Before staining, Fc receptors were blocked using anti-CD16/32 Ab (BD Biosciences). Then, cells were incubated with Abs for cell surface staining. Spleen NK and CD8 T cells were determined by using a FACS Calibur flow cytometer (Becton Dickinson). For cell surface staining, mouse splenocytes were incubated with either fluorescein isothiocyanate (FITC)-conjugated anti-mouse CD3e mAb, APC-conjugated anti-mouse NKp46 mAb (52, 53), and FITC-conjugated anti-mouse CD11b mAb, or FITC-conjugated anti-mouse CD3e mAb, APC-conjugated anti-mouse CD8 mAb, PE-conjugated anti-mouse CD122 mAb, and FITC-conjugated anti-mouse CD44 mAb. Data were analyzed using FlowJo software (BD Biosciences).

### Statistical Analysis

Statistical analyses were performed using Mann–Whitney test and Kruskal–Wallis test with Dunn’s post-test (GraphPad Prism Software). Data are expressed as mean ± SEM. A *p*-value <0.05 was considered significant (*p < 0.05; **p < 0.01; ***p < 0.001).

## Results

### Strategy to Selectively Block IL-15 Recruitment of IL-2Rβ/γc While Retaining IL-15Rα Binding

We sought to selectively inhibit the action of IL-15 through the trimeric IL-15Rα/IL-2Rβ/γc receptor to assess whether the pro-inflammatory property of IL-15 is due to binding to its specific receptor chain, IL-15Rα. To this end, we designed NANTIL-15 that retains binding to IL-15Rα while not recruiting the IL-2Rβ transducing receptor chain.

We confirmed that NANTIL-15’s binding parameters to IL-15Rα were comparable to that of wild-type IL-15, whereas its binding properties to IL-2Rβ chain was completely impaired (**Figure 1B**) (54). We also observed that NANTIL-15 was not capable of binding to immobilized recombinant dimeric Fc-IL-2Rβ/γc receptor, suggesting that blocking the binding to IL-2Rβ chain is sufficient to fully impair the binding to the dimeric receptor (**Figure 1C**). We further investigated the IL-15Rα/IL-2Rβ/γc binding selectivity of NANTIL-15 by using Kit225 cells, expressing the three receptor chains and thus both trimeric IL-15Rα/IL-2Rβ/γc and dimeric IL-2Rβ/γc forms (48), and also Kit225-15RαKO cells in which *il15rα* gene was invalidated and therefore expressing only the dimeric IL-2Rβ/γc receptor (48). According to our previous results, NANTIL-15 binding was detected at the surface of Kit225 but not of Kit225-15RαKO cells, whereas Fc-IL-15 binding was detected at the surface of both cells (**Figure 1D**). Taken together, these data show that abrogating the recruitment of IL-15 to IL-2Rβ is sufficient to fully impair the binding to dimeric IL-2Rβ/γc receptor, while retaining the binding to IL-15Rα.

### NANTIL-15 Does Not Affect IL-15 Signaling Through the Dimeric IL-2Rβ/γc Receptor

**Figure 2:**
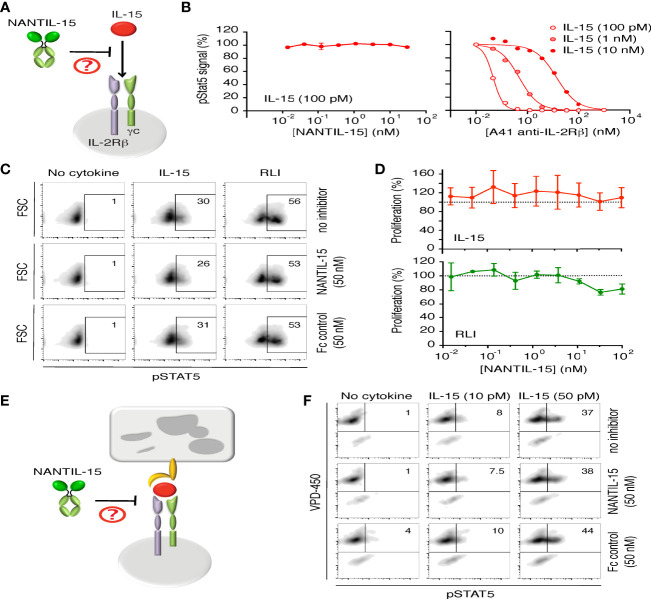
NANTIL-15 does not affect IL-15’s signaling through dimeric IL-2Rβ/γc receptor. **(A)** Diagram showing NANTIL-15 acting on the signaling of IL-15 through the dimeric IL-2Rβ/γc receptor. **(B)** Left panel: Effect of increasing concentrations of NANTIL-15 on IL-15 (100 pM)-induced Stat5 phosphorylation within Kit225-15RαKO cells by AlphaScreen. Right panel: Inhibition of IL-15 (100 pM, 1 nM, and 10 nM)-induced Stat5 phosphorylation within Kit225-15RαKO cells by increasing concentrations of blocking A41 anti-IL-2Rβ antibody by AlphaScreen. **(C)** Effect of NANTIL-15 or Fc control on IL-15 (50 pM)- or RLI (100 pM)-induced Stat5 phosphorylation within Kit225-15RαKO cells by flow cytometry. Number indicated the percentage of pStat5 positive cells. **(D)** Proliferation of Kit225-15RαKO cells in response to fixed concentrations of IL-15 (upper panel) or RLI (lower panel) in the presence of increasing concentrations of NANTIL-15. **(E)** Diagram showing NANTIL-15 acting on IL-15 *trans*-presentation. **(F)** Flow cytometric analysis of pStat5 expression within VPD-450 labeled Kit225 responding cells following 1 h coculture with wt.IL-15Rα HEK-293 expressing cells loaded with IL-15 (10 or 50 pM) in the presence or the absence of NANTIL-15 (50 nM). Number indicated the percentage of pStat5-positive cells. All data are representative of at least three separate experiments.

Then, we evaluated the functional inhibitory efficacy and the receptor specificity of NANTIL-15 (**Figure 2A**). First, we confirmed that NANTIL-15 was not capable of inducing either Stat5 phosphorylation or proliferation of Kit225-15RαKO cells, expressing only the dimeric IL-2Rβ/γc receptor (**Figures 2B**, *left panel*, **C**, **D**). As expected, NANTIL-15 was unable to inhibit IL-15-induced Stat5 phosphorylation in Kit225-15RαKO cells, whereas A41 anti-IL-2Rβ antibody was able to effectively inhibit this signal (**Figures 2B, C**). Then, we took advantage of the RLI fusion molecule that selectively signals through the dimeric IL-2Rβ/γc receptor, regardless of the expression of IL-15Rα. On Kit225-15RαKO cells, Stat5 phosphorylation and cell proliferation induced by a fixed concentration of IL-15 or RLI were challenged by increasing concentrations of NANTIL-15. NANTIL-15 did not affect these signals in either condition (**Figures 2C, D**). Taken together, these data show that NANTIL-15 does not affect IL-15 signaling through the dimeric IL-2Rβ/γc receptor.

Then, we wondered whether NANTIL-15 could affect IL-15 *trans*-presentation (**Figure 2E**). NANTIL-15 or Fc-IL-15 was loaded onto HEK-293 cells stably transfected with IL-15Rα and co-cultured in the presence of responding Kit225 cells (51). No Stat5 phosphorylation was observed when Kit225 cells were in the presence of NANTIL-15-loaded HEK-293-IL-15Rα cells, whereas pStat5 was detected when Kit225 cells were co-cultured with Fc-IL-15-loaded HEK-293-IL-15Rα cells (data not shown). These data indicate that NANTIL-15 does not signal in *trans*.

It has been shown that IL-15 *trans*-presentation requires the association of IL-15 with IL-15Rα inside the presenting cells, prior from emerging to the cell surface (14), and this mode of action is the dominant mechanism by which IL-15 delivers its signal in physiological conditions (55). To test whether, NANTIL-15 could inhibit IL-15 *trans*-presentation, wild-type IL-15 was first loaded to HEK-293-IL-15Rα and co-cultured with responding Kit225 cells, in the presence or in the absence of NANTIL-15. We observed that IL-15 *trans*-presentation was not inhibited by the presence of NANTIL-15, indicating that NANTIL-15 was not able to chase IL-15 bound to IL-15Rα (**Figure 2F**). Thus, NANTIL-15 does not affect IL-15’s action through the dimeric IL-2Rβ/γ receptor either in *cis* and in *trans*.

### NANTIL-15 Selectively Inhibits IL-15 Signaling Through the Trimeric IL-15Rα/IL-2Rβ/γc Receptor

**Figure 3:**
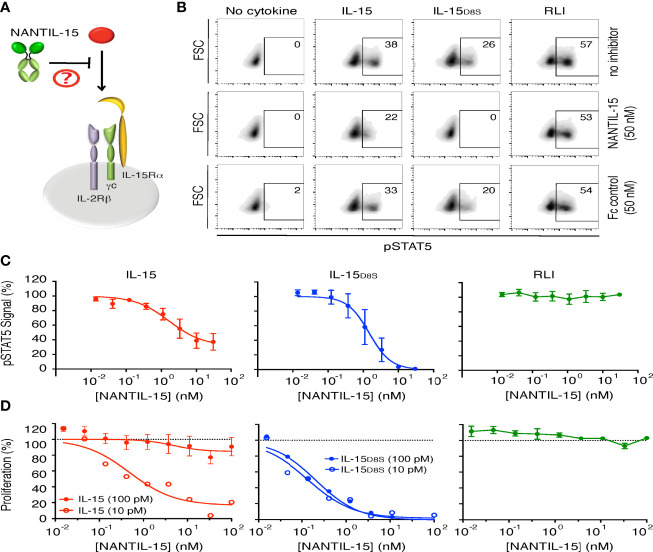
NANTIL-15 selectively inhibits IL-15’s signaling through trimeric IL-15Rα/IL-2Rβ/γc receptor. **(A)** Diagram showing NANTIL-15 acting on the signaling of IL-15 through the trimeric IL-15Rα/IL-2Rβ/γc receptor. **(B)** Inhibition of IL-15 (50 pM)-, IL-15D8S (300 pM)-, and RLI (100 pM)-induced Stat5 phosphorylation by 50 nM of NANTIL-15 or Fc control within Kit225 cells. Number indicated the percentage of pStat5-positive cells. **(C)** Analysis of relative pStat5 levels in Kit225 cells in response to fixed concentrations of IL-15 (100 pM), IL-15_D8S_ (300 pM), or RLI (3 nM) in the presence of increasing concentrations of NANTIL-15, by AlphaScreen technology. **(D)** Inhibition of IL-15 (10 or 100 pM), IL-15_D8S_ (10 or 100 pM), or RLI (100 pM) induced Kit225 cell proliferation by increasing concentrations of NANTIL-15. Data are representative of at least three separate experiments.

We further evaluated the inhibitory efficacy and the receptor specificity of NANTIL-15 using Kit225 cells expressing both types of receptors, including the trimeric IL-15Rα/IL-2Rβ/γc receptor (**Figure 3A**). Interestingly, NANTIL-15 partially inhibited IL-15-induced Stat5 phosphorylation, reaching a plateau at 60% of inhibition with an IC50 of 1 nM (**Figures 3B, C**). These results suggested that NANTIL-15 is capable of selectively impairing IL-15’s signaling through the trimeric IL-15Rα/IL-2Rβ/γc receptor without affecting that through the dimeric IL-2Rβ/γc receptor. To confirm that hypothesis, we took advantage of the IL-15_D8S_ mutant that is only able to signal through the trimeric IL-15Rα/IL-2Rβ/γc receptor (48). A fixed concentration of IL-15_D8S_ was submitted to increasing concentrations of NANTIL-15 on Kit225 cells. NANTIL-15 fully abrogated IL-15_D8S_-induced pStat5 signal with an IC50 of 1 nM comparable to that of wild-type IL-15 (**Figure 3C**). Cell proliferation assays confirmed these observations, with an IC50 below 1 nM (**Figure 3D**). According to our previous results, NANTIL-15 was ineffective to inhibit pStat5 and the proliferation of Kit225 cells when stimulated with RLI, confirming that NANTIL-15 does not inhibit IL-15 signaling through the dimeric IL-2Rβ/γc receptor (**Figures 3C, D**). Taken together, these data show that NANTIL-15 is a selective potent antagonist of IL-15 acting in *cis* through the trimeric IL-15Rα/IL-2Rβ/γc receptor without affecting its *cis* action through the dimeric IL-2Rβ/γc receptor. Thus, NANTIL-15 inhibitory property is closely related to the expression of IL-15Rα on the cell surface of target cells.

### NANTIL-15 Does Not Affect NK and CD8 T-Cell Population *In Vivo*

**Figure 4:**
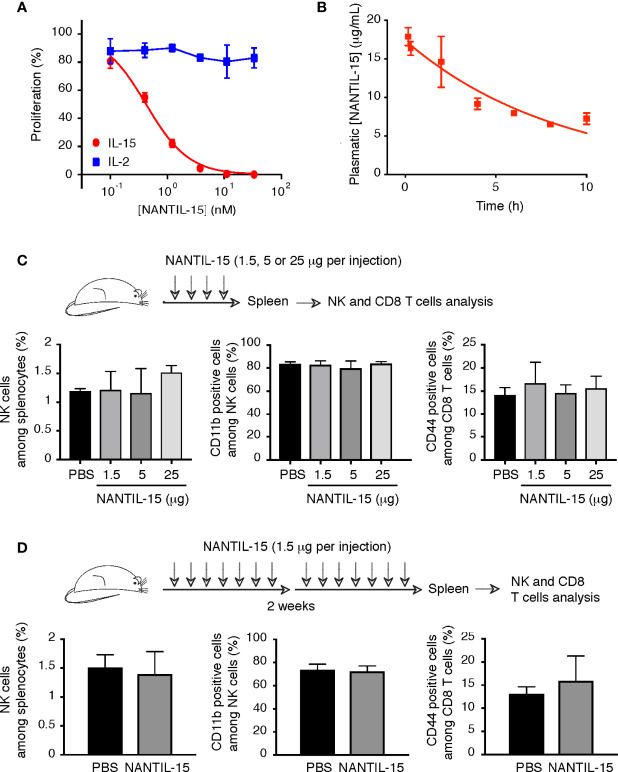
NANTIL-15 administration does not affect NK and memory CD8 T-cell homeostasis. **(A)** Inhibition of IL-15 (10 pM) or IL-2 (100 pM) induced CTLL-2 cell proliferation by increasing concentrations of NANTIL-15. **(B)** The half-life of NANTIL-15 was evaluated in C57BL/6 mice after a single intravenous injection of 10 µg of the molecule. At each time point (n = 3), the blood was collected. NANTIL-15 concentration in the serum was determined by ELISA. **(C)** Analysis of total splenic NK cells, mature (CD11b^+^) NK cells, and CD44^+^CD8 T cells following i.p. injection of 1.5, 5, or 25 μg of NANTIL-15 (every day for 4 days). **(D)** Analysis of total splenic NK cells, mature (CD11b^+^) NK cells, and CD44^+^CD8 T cells following repeated i.p. injections of 1.5 μg of NANTIL-15 (every day for 2 weeks). All data are representative of at least two separate experiments (n = 3, each experiment).

To test the efficacy of NANTIL-15 to inhibit IL-15’s action in mice, we first took advantage of the CTLL-2 murine cell line, known to express murine IL-15Rα, IL-2Rα, IL-2Rβ, and γc receptor chains. As observed for human cells, NANTIL-15 was also able to inhibit IL-15-induced signal on CTLL-2 cells, whereas IL-2-induced signal remained unaffected (**Figure 4A**). In mice, we first determined NANTIL-15’s plasmatic half-life, estimated at around 6 h (**Figure 4B**). Then, to evaluate the relative impact of the administration of NANTIL-15 to IL-15’s dependent cells, such as NK and CD44^+^CD8 T cells (6), increasing doses (from 1.5 to 25 μg) of NANTIL-15 were injected in 4 consecutive days. No effect on NK and CD8 T-cell populations could be observed even at the highest dose (25 μg for a total amount of 100 μg) (**Figure 4C**). We also observed that repeated injections of 1.5 μg of NANTIL-15 every day during 2 weeks (total of 21 μg) did not affect either of these populations (**Figure 4D**). Moreover, NK cell maturation, revealed by the CD11b marker, was not affected either. Thus, NANTIL-15 injections do not disturb NK and CD8 T-cell populations in healthy mice.

### NANTIL-15 Reduces Local Inflammation Without Any Impact on the Periphery

**Figure 5:**
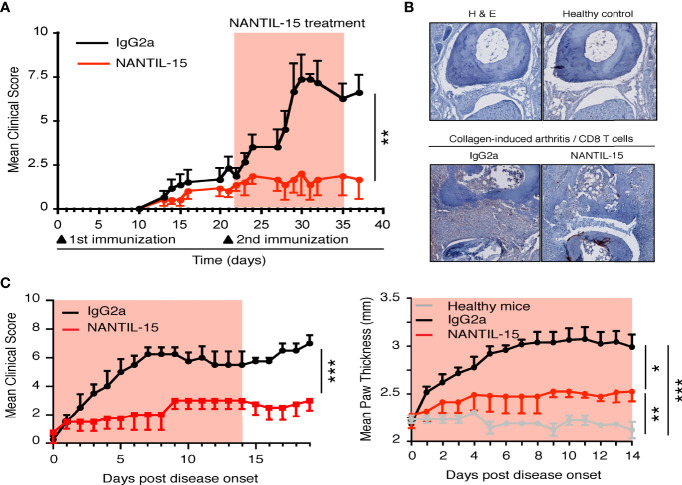
NANTIL-15 reduces local inflammation in a collagen-arthritis mouse model. **(A)** NANTIL-15 reduces the signs of inflammation in a collagen-induced arthritis mouse model. Mice were immunized with collagen and challenged 21 days later with a second injection of collagen. One group of mice was treated 1 day following the second immunization with NANTIL-15 (5 μg/mouse/day), the other by the negative control IgG2a for 14 days. The clinical score was monitored for the duration of the experimental period (the light red squares indicate the period of treatment with NANTIL-15). Means clinical score ± SEM are given. **(B)** NANTIL-15 treatment reduces articular inflammation and joint destruction. Histological examination of ankle joints. After development of the first symptoms of arthritis, joints from NANTIL-15- and IgG2a-treated animals (n = 6) were removed, formalin-fixed, and decalcified, and H&E stains were prepared. Representative sections of ankles from paws with a disease score of 2 on day 7 after disease onset are shown (magnification 40×). Whereas IgG2a-treated animals show severe joint cartilage erosion and joint space narrowing (left panel), joints from NANTIL-15-treated animals show a relative normal morphology (right panel). **(C)** In a second collagen-induced arthritis mouse model experimental design, mice were immunized with collagen and challenged 21 days later with a second injection of collagen. Mice were synchronized when the first symptoms of the disease appeared and divided into two groups. One group was treated with NANTIL-15 (1.5 μg/mouse/day), the other by the negative control IgG2a for 14 days. The clinical score (left panel; Mann–Whitney test, ***IgG2a vs. NANTIL-15) and paw thickness (right panel; Kruskal–Wallis test with Dunn’s post-test, *IgG2a vs. NANTIL-15, ***IgG2a vs. healthy, **NANTIL-15 vs. healthy) were monitored during treatment and for a period of 2 weeks (the light red squares indicate the period of treatment with NANTIL-15). Means clinical score and paw thickness ± SEM are given.

IL-15 overexpression has been observed in human and murine model of rheumatoid arthritis. Thus, different strategies have been developed to block IL-15’s action reducing the severity of the disease (18). To further understand how IL-15 functions in this pathological condition, and which mode of action needs to be targeted, the efficacy of NANTIL-15 to attenuate inflammation was evaluated in the collagen-induced arthritis (CIA) model, in which IL-15 has been shown to participate to the inflammatory process (39). We performed two experimental models of CIA. In the first, mice were exposed to collagen twice 2 weeks apart. Treatment with NANTIL-15 began the day after the second immunization (day 22), when mice had already developed signs of inflammation for about 10 days. By measuring the clinical scores daily, we observed that mice treated with NANTIL-15 did not develop severe arthritis compared to those treated with Ig2a isotype control (**Figure 5A**). Histological examination of joints of NANTIL-15-treated animals revealed reduction in articular inflammation compared to controls. Immunohistology also showed that infiltration by CD8 T cells was reduced in mice treated with NANTIL-15 compared to controls (**Figure 5B**). In the second experimental design, NANTIL-15 was administrated every day for 2 weeks when the first inflammatory symptoms appeared. By measuring daily the clinical scores and paw thickness, we observed that NANTIL-15 treatment significantly reduced the inflammatory signs, compared to controls (mice treated with Ig2a isotype control) (**Figure 5C**). Interestingly, 5 days after the last injection of NANTIL-15, no increase in clinical signs was observed (**Figure 5C**, left panel).

**Figure 6:**
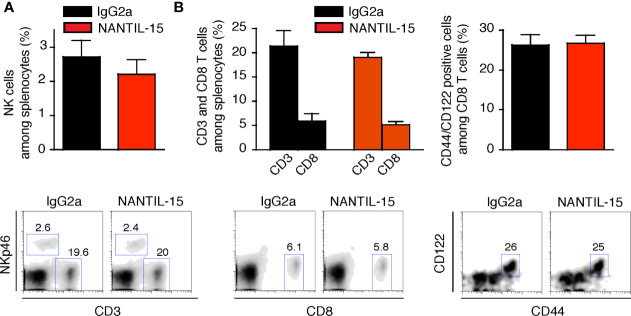
NANTIL-15 treatment of collagen-induced arthritic mice does not affect peripheric homeostasis. **(A)** Analysis of total splenic NK cells and **(B)** total splenic CD3, CD8, and CD44^+^ CD122^+^CD8 T cells after 2 weeks of treatment. All data are representative of at least two to three separate experiments (n = 6).

**Figure 7:**
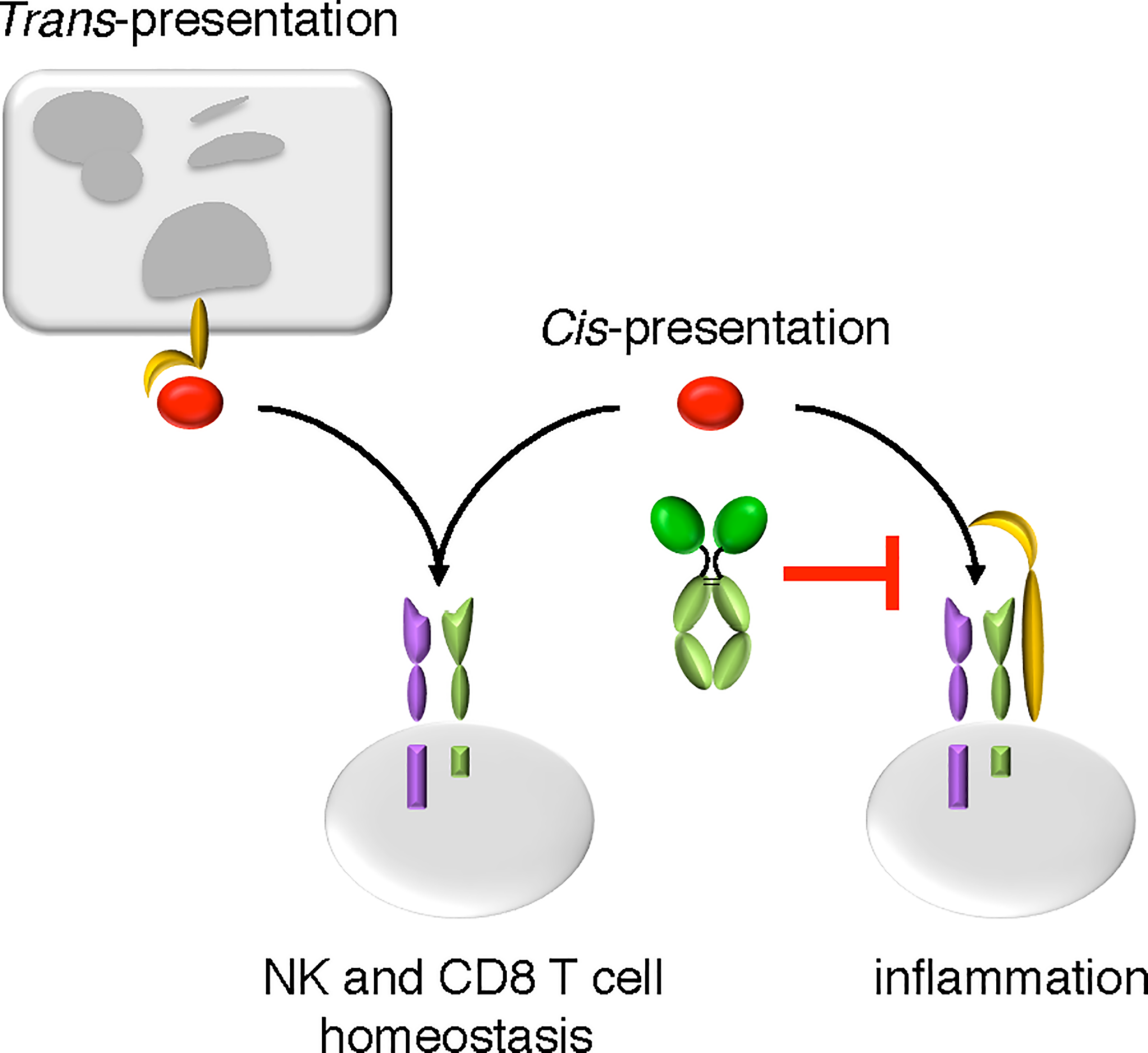
The mode of action of NANTIL-15. NANTIL-15 selectively inhibits the action of IL-15 *via* the trimeric IL-15Rα/IL-2Rβ/γc receptor, leaving its action *via* the dimeric IL-2Rβ/γc receptor or in *trans*-presentation intact. By reducing the clinical signs of inflammation without any impact on NK and CD8 T cells homeostasis, the action of NANTIL-15 indicates that IL-15-induced inflammation acts through the trimeric IL-15Rα/IL-2Rβ/γc receptor.

Splenic NK and CD8 T-cell populations were analyzed to evaluate the impact of NANTIL-15 treatment in the periphery of arthritic mice. No significant difference was observed between NANTIL-15-treated and control mice in terms of total NK cells, CD3, CD8, and CD44^+^CD122^+^CD8 T cells in the spleen (**Figures 6A, B**). Taken together, these results show that NANTIL-15 treatment reduces inflammation locally without any impact on NK and CD8 T cell homeostasis (**Figure 7**), indicating that inhibition of IL-15’s action through the trimeric IL-15Rα/IL-2Rβ/γc receptor is sufficient to reduce inflammation while leaving IL-15’s target cell homeostasis unaffected.

## Discussion

In this study, we generated an IL-15-derived molecule (called NANTIL-15 for New ANTagonist of IL-15) to selectively block the action of IL-15 through the trimeric IL-15Rα/IL-2Rβ/γc receptor. For that purpose, we targeted the molecular interface of IL-15 facing IL-2Rβ in order to prevent the recruitment of the latter while maintaining the binding to IL-15Rα. Residues N65 and L69 of IL-15 were previously shown to be crucial for IL-2Rβ binding (47, 48) and were therefore mutated in NANTIL-15. Knowing that IL-2Rβ provides the primary anchor for IL-2Rβ/γc receptor binding, we postulated that NANTIL-15 would not be able to bind the dimeric IL-2Rβ/γc receptor (48). We confirm this hypothesis by SPR and flow cytometry, as NANTIL-15 was not able to bind the isolated IL-2Rβ chain nor the dimeric IL-2Rβ/γc receptor. Thus, preventing IL-2Rβ binding is sufficient to completely avoid the binding of IL-15 to the dimeric receptor. As a result, NANTIL-15 does not affect the signaling of free soluble IL-15 or IL-2 through this receptor. Furthermore, IL-15 *trans*-presentation, the dominant mechanism by which IL-15 delivers its signal *in vivo* (55, 56), was also not affected. This could be explained by the fact that during that process, IL-15 binds to IL-15Rα inside the cells before emerging at the cell surface (14), thus avoiding binding of NANTIL-15 to IL-15Rα. IL-15Rα expression is highly regulated and under homeostatic conditions; IL-15 target cells, such as NK and CD8 T cells, express predominantly the IL-2Rβ/γc receptor. Therefore, repeated injections or high doses of NANTIL-15 administrated to mice did not affect the NK and CD44^+^CD8 T-cell populations. Indeed, IL-15Rα-deficient NK cells survive in normal mice (57) and IL-15Rα expression on CD8+ T cells is not required for T cell memory (58). However, when IL-15Rα was expressed together with IL-2Rβ and γc with the ability to form a high-affinity trimeric receptor, NANTIL-15 was able to effectively block IL-15 signaling. This effect is even more pronounced on cells expressing a large majority of IL-15Rα/IL-2Rβ/γc receptor on their surface. Overall, we demonstrated that NANTIL-15, by retaining IL-15Rα binding without recruiting IL-2Rβ, is a selective inhibitor of IL-15 acting through the IL-15Rα/IL-2Rβ/γc receptor without affecting its action through the dimeric IL-2Rβ/γc receptor. This property allowed us to investigate whether the inflammatory property of IL-15 depends on its binding to its private IL-15Rα chain.

It is now well established that IL-15 is involved in inflammatory states. Overexpression of IL-15 has been shown to be involved in several autoimmune disorders including rheumatoid arthritis (RA) (20, 27), psoriasis (41), inflammatory bowel diseases (59), celiac disease (25, 60), lupus (21), vitiligo (61), alopecia areata (62), and multiple sclerosis (63, 64). IL-15 protein has been detected in the synovial fluids and synovial membranes of patients with active RA (65). Synovial fluids of RA subjects were found to promote activation and chemoattraction of T cells, which were partially blocked by anti-IL-15 antibodies. Accordingly, IL-15-activated T cells from RA patients stimulated monocytes and macrophages to produce TNFα *in vitro* in a cell-contact-dependent manner (20). Thus, overproduction of IL-15 by lining layer macrophages, fibroblast-like synoviocytes (FLS), and endothelial cells is thought to participate to the pathogenesis of RA by inducing T-cell activation and proliferation (27, 66). In addition to their IL-15 overproduction, RA FLS express the specific IL-15Rα and IL-2Rβ and γc chains and are therefore able to express the dimeric and the trimeric receptor of IL-15. Furthermore, it is interesting to note that unstimulated FLS express IL-2Rβ and γc receptor chains, but in the presence of TNFα, IL-15 and IL-15Rα are upregulated, resulting in autocrine proliferation of FLS (67). Thus, IL-15 and IL-15Rα appear to play an important role in the pathogenesis of RA.

To regulate the action of IL-15 at the protein level, natural variants of IL-15, IL-15ΔE6, and IL-15ΔE7 have been reported in mouse gut, being natural antagonists of IL-15 function. Indeed, they exhibited an inhibitory effect on IL-15-mediated CTLL-2 and T-cell proliferation (68, 69). The IL-15 isoform lacking exon-6, IL-15ΔE6, is generated by alternative splicing in activated immune cells, including macrophages and B cells. IL-15ΔE6 does not induce the phosphorylation of Stat5 while retaining a capacity to bind IL-15Rα and interfere with the binding of wild-type IL-15 to IL-15Rα (69). In different mouse model of skin inflammation, IL-15ΔE7 inhibited abrasion-induced keratinocyte proliferation, skin thickening, cytokine production, and neutrophil infiltration (70). Overexpression of IL-15ΔE6 improved EAE symptoms of mice (69). Thus, such endogenous IL-15 antagonistic isoforms may help interfere with and protect against IL-15-induced inflammation.

As IL-15 has been shown to be involved early in the cascade that ultimately leads to inflammatory disease (19), IL-15 is presented as a potentially interesting target for the disease. Different strategies have been developed to inhibit the action of IL-15. Based on the observations of Pettit et al., Strom’s group designed an IL-15 mutant (IL-15Q101D/Q108D) having lost the capacity to interact with the γc receptor chain and that competitively blocks IL-15-triggered cell proliferation (29, 38). This molecule, called CRB-15, inhibited the incidence and severity of arthritis in CIA mouse model mice (39). The anti-IL-15 Ab from Amgen AMG-714 or HuMax-IL15 has shown efficacy in a xenograft model of human psoriasis, RA, and coeliac disease (20, 45). CRB-15 and AMG714 Ab, both by targeting the interface of IL-15 facing γc, should theoretically broadly inhibit the action of IL-15, since IL-2Rβ recruitment remains unaffected.

By targeting the IL-2Rβ receptor chain, the Hu-Mik-β1 Ab (30) inhibits IL-2- and IL-15-induced activation and proliferation of resting NK and T cells expressing the dimeric IL-2Rβ/γ_c_ receptor. It does not block the action of IL-2 on cells expressing the trimeric IL-2 receptor, such as regulatory T cells. Thus, it is used in clinical trials in patients with autoimmune diseases, in particular refractory celiac disease. The efficacy of TM-β1 (the anti-mouse IL-2Rβ equivalent of Hu-Mik-β1) was evaluated in a mouse model of celiac disease using transgenic mice that express IL-15 in intestinal cells using an enterocyte-specific T3b promoter to drive the transgene (71). TMβ1 administration was associated with the complete reversal of the macroscopic and microscopic pathological changes in the intestine of T3b-h IL-15 Tg mice. However, this anti-IL-2Rβ also inhibits IL-15 *trans*-presentation, making it unclear which mode of action of IL-15 is involved in inflammatory conditions. Some strategies also aimed to interfere with IL-15Rα binding such as a soluble form of IL-15Rα (40) and peptides targeting IL-15Rα chain at the IL-15 binding interface (33, 72). Both approaches prevent the binding of IL-15 to IL-15Rα. Soluble IL-15Rα captures soluble IL-15 prior to binding to membrane-bound IL-15Rα, but the IL-15.IL-15Rα complex formed is known to have a greater potency than IL-15 to activate T cells. The P8 peptide binds to IL-15Rα and prevents IL-15 binding. However, its binding capacity (IC50 higher than 10 μM) is much lower than that of NANTIL-15 (IC50 below the nM range). Finally, rather than being specific to IL-15 alone, a series of broad-spectrum inhibitors are being developed to block multiple γc cytokines by targeting the γc receptor chain. The efficacy of BNZ multicytokine inhibitors (32, 45) is under evaluation in alopecia areata and coeliac disease with promising results (46). However, such approach does not allow to discriminate precisely which cytokine is involved and even less which molecular mode of action of IL-15 (*cis* or *trans*) could be incriminated in the inflammatory conditions.

IL-15 and IL-15Rα are required for the homeostasis and survival of not only NK and memory CD8 T cells but also NK-T cells and a subset of IELs (6, 12). This action is mediated by IL-15 *trans*-presentation. In addition, IL-15 may act on other cell types such as CD4 T cells (73, 74), monocytes/macrophages (75, 76), neutrophils (77), or B cells (78). Further studies need to be evaluated to determine the cells targeted by NANTIL-15 in the CIA mouse model. Preliminary evidence suggests that certain cells expressing the trimeric IL-15Rα/IL-2Rβ/γc receptor might be good candidates. Indeed, in RA, Ruckert and colleagues suggested that excess IL-15 may stimulate monocytes/macrophages through the trimeric receptor to activate the autoreactive CD4^+^ T cells (76). In addition, IL-15 mutant/Fcγ2a (CRB-15), a molecule equivalent to NANTIL-15 but with an IL-15 mutein impaired for γc recruitment, was tested on human RA FLS. The authors demonstrated that the persistent activation and proliferation of RA FLS are related to an IL-15 autocrine loop, acting through the trimeric IL-15Rα/IL-2Rβ/γc receptor, which can be inhibited by the IL-15 mutant/Fcγ2a (67). IL-15 could also mediate neutrophil migration into inflamed tissues. Interestingly, neutrophil migration was completely inhibited by soluble IL-15Rα (79), confirming that neutrophils express a functional trimeric IL-15Rα/IL-2Rβ/γc receptor (80). Taken together, RA FLS, macrophages, and neutrophils among other cells may be targeted by NANTIL-15, which should be evaluated in future studies.

Overall, our results demonstrated that selectively avoiding IL-15 signaling through the trimeric IL-15Rα/IL-2Rβ/γc receptor is important for reducing inflammation in the collagen-induced arthritis model. They support the hypothesis that the deleterious action of IL-15 in RA is directed by free soluble IL-15 acting in *cis* through the trimeric IL-15Rα/IL-2Rβ/γc receptor rather than by *trans*-presented IL-15 or soluble IL-15.IL-15Rα complexes. Finally, we demonstrated that NANTIL-15, by its design, acts locally at the site of inflammation without any detectable impact on IL-15-dependent cell homeostasis. This study provides evidence that selective targeting IL-15Rα chain is sufficient to reduce inflammation while limiting systemic toxicity.

## Data Availability Statement

The original contributions presented in the study are included in the article. Further inquiries can be directed to the corresponding author.

## Author Contributions

Conceptualization: EM. Methodology: DM, MM, AQ, and EM. Formal analysis: DM, MM, SM, AQ, and EM. Investigation: DM, MM, IB, SM, DK, AQ, and EM. Funding acquisition: AQ, YJ, and EM. Writing—original draft: EM. Writing—review and editing: AQ and EM. Supervision: EM. All authors contributed to the article and approved the submitted version.

## Conflict of Interest

The authors declare that the research was conducted in the absence of any commercial or financial relationships that could be construed as a potential conflict of interest.

## Publisher’s Note

All claims expressed in this article are solely those of the authors and do not necessarily represent those of their affiliated organizations, or those of the publisher, the editors and the reviewers. Any product that may be evaluated in this article, or claim that may be made by its manufacturer, is not guaranteed or endorsed by the publisher.
